# Evolutional selection of a combinatorial phage library displaying randomly-rearranged various single domains of immunoglobulin (Ig)-binding proteins (IBPs) with four kinds of Ig molecules

**DOI:** 10.1186/1471-2180-8-137

**Published:** 2008-08-13

**Authors:** Hua Yang, Jie Cao, Lian-Qing Li, Xia Zhou, Qiu-Li Chen, Wen-Ting Liao, Zong-Mei Wen, Shao-Hua Jiang, Rong Xu, Jian-An Jia, Xin Pan, Zhong-Tian Qi, Wei Pan

**Affiliations:** 1 Department of Microbiology and Immunology, Shan Xi Medical University, Tai-yuan, 030001, PR China; 2 Department of Microbiology, State Key Laboratory of Medical Immunology, Second Military Medical University, Shanghai 200433, PR China; 3 Center of clinical diagnosis, Shan Xi province, Tai-yuan, 030012, PR China

## Abstract

**Background:**

Protein A, protein G and protein L are three well-defined immunoglobulin (Ig)-binding proteins (IBPs), which show affinity for specific sites on Ig of mammalian hosts. Although the precise functions of these molecules are not fully understood, it is thought that they play an important role in pathogenicity of bacteria. The single domains of protein A, protein G and protein L were all demonstrated to have function to bind to Ig. Whether combinations of Ig-binding domains of various IBPs could exhibit useful novel binding is interesting.

**Results:**

We used a combinatorial phage library which displayed randomly-rearranged various-peptide-linked molecules of D and A domains of protein A, designated PA(D) and PA(A) respectively, B2 domain of protein G (PG) and B3 domain of protein L (PL) for affinity selection with human IgG (hIgG), human IgM (hIgM), human IgA (hIgA) and recombinant hIgG1-Fc as bait respectively. Two kinds of novel combinatorial molecules with characteristic structure of PA(A)-PG and PA(A)-PL were obtained in hIgG (hIgG1-Fc) and hIgM (hIgA) post-selection populations respectively. In addition, the linking peptides among all PA(A)-PG and PA(A)-PL structures was strongly selected, and showed interestingly divergent and convergent distribution. The phage binding assays and competitive inhibition experiments demonstrated that PA(A)-PG and PA(A)-PL combinations possess comparable binding advantages with hIgG/hIgG1-Fc and hIgM/hIgA respectively.

**Conclusion:**

In this work, a combinatorial phage library displaying Ig-binding domains of protein A, protein G, or protein L joined by various random linking peptides was used to conducted evolutional selection *in vitro* with four kinds of Ig molecules. Two kinds of novel combinations of Ig-binding domains, PA(A)-PG and PA(A)-PL, were obtained, and demonstrate the novel Ig binding properties.

## Background

Bacterial immunoglobulin (Ig)-binding proteins (IBPs) are cell-anchored which can bind to specific sites on Ig of the host and mediate pathogenicity in host [1]. Protein A of *Staphylococcus aureus *(SpA), protein G of group C and G *streptococci *(SpG), and protein L of *Finegoldia magna *formerly *Peptostreptococcus magnus *are three well-defined IBPs. Although the precise functions of these molecules are not fully understood, it is thought that they play an important role in pathogenicity of bacteria. SpA comprises 524 amino acid residues with a molecular weight of 57 KD. The extracellular part of SpA contains a tandem repeat of five highly homologous IgG-binding domains designated (from the N terminus) E, D, A, B and C, each of which contains about 58 amino acid residues. The overall structures of these domains are all up-down three α-helixes and all five domains of SpA show Ig-binding abilities [2-4]. SpG (about 63 KD) is composed of 594 amino acid residues, containing 3 highly homologous Ig-binding domains identified as B1, B2 and B3 [5]. Each domain of SpG consists of two pairs of antiparallel β-sheets connected by a single α-helix [6,7]. Protein L is a 95 KD protein and contains 719 amino acid residues, with the Ig-binding activity located in five homologous repeats, Bl-B5, each comprising 72–76 amino acid residues. The fold of the Ig-binding domains of protein L is similar to that of the domains of SpG [8,9].

Both SpA and SpG show high affinity for the interface between the second constant region of heavy chain (CH2) and CH3 domains (CH2γ–CH3γ) of IgG crystallizable fragment (Fc) [10]. In addition, SpA can bind to fragment of antigen binding (Fab) of a subset of Igs with variable region of heavy chains belonging to human VHIII family, so SpA is capable of binding to other types of Ig molecules besides IgG [11,12]. In contrast to SpA, SpG binds to Fab with the first constant region of Ig γ chain (CH1γ), so SpG can bind only to IgG [13]. Protein L has been shown to bind to κ light chains of Ig, so it can bind to all types of Ig molecules that contain a kappa light chain [14,15].

Some hybrid IBPs, like protein AG, Protein LG and Protein LA had been constructed [16-18], and were shown to sustain Ig-binding property of parental proteins and broaden Ig-binding spectra. Protein AG was shown to possess higher affinity for IgG than SpA or SpG. In addition to the enhanced affinity for IgG and IgG-Fc, protein LA showed enhanced affinity for Ig Fab or majority of human single chain variable antibody fragment (scFv) carrying the κ light-chain variable domain or expressing the VHIII determinant. It suggested complementary effect of Ig-binding in these hybrid IBPs.

The single domains of SpA, SpG and protein L were all demonstrated to have function to bind to Ig [17,19-21]. Whether combinations of Ig-binding domains of various IBPs could exhibit useful novel binding remain interesting. In this study, we used the single Ig-binding domains of SpA, SpG and protein L as basic functional units to construct a combinatorial phage library displaying randomly-rearranged molecules joined by various random linking peptides, and conducted evolutional selection *in vitro* with four kinds of Ig molecules as bait. Two kinds of novel combinations of Ig-binding domains, PA(A)-PG and PA(A)-PL, were obtained, and might represent improved Ig-binding properties.

## Results

### Distribution of various fragment sizes displayed by phage library and post-selection populations

**Figure 1:**
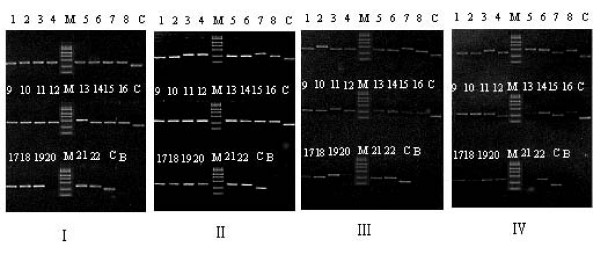
**Detection of inserted fragments of phage clones in each round of hIgG selection library by PCR**. PCR products were analyzed by electrophoresis in 1.2% agarose gel and detected by staining with ethidium bromide. No. 1 to 22: randomly picked phage clones; M: DL2000 Marker; C: positive control (pCANTAB5S vector); B: negative control (blank culture medium); I, II, III, IV: the first to the fourth round of selection respectively.

**Figure 2:**
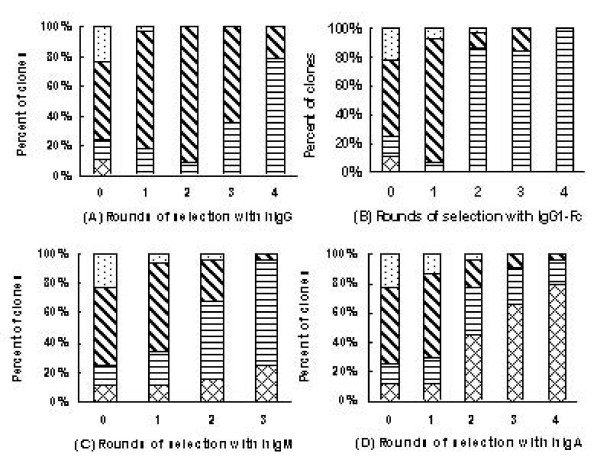
**Proportion of phage clones with different sizes of inserted fragments in 22 phage clones after each round of selection with four Ig molecules respectively (A-D)**. : phage clones with no inserted fragment; : phage clones displaying one domain of combinatorial Ig-binding molecules; : phage clones displaying two domains of combinatorial Ig-binding molecules; : phage clones displaying three domains of combinatorial Ig-binding molecules.

To evaluate the Ig affinity selection efficacy, some markers including phage library binding capacity, output/input ratio of phages, distribution of various fragment size etc. were measured. The library binding capacity and output/input ratio did not correspond well with the affinity selection (data not shown). We found the distribution of fragment sizes changed remarkably during the selection (Fig. 1). As figure 2 shows, the proportion of phage clones displaying two domains and three domains in original library was 22%, then increased dramatically and reached 80%–100% after four rounds of selection with hIgG and recombinant hIgG1-Fc (Fig. 2A, 2B). In hIgM and hIgA selection, the proportion of phage clones displaying two domains and three domains also increased remarkably from 22% to 98% and 22% to 96% respectively after three or four rounds of selection (Fig. 2C, 2D). These results corresponded well with our previous experiment that also indicated that along with the rounds of selection, the proportion of phage clones displaying large fragments increased [22], and it might represent effective selection.

### Analyses of inserted fragments of the post-selection populations

In the fourth post-selection population selected with hIgG or hIgG1-Fc, twenty phage clones were randomly chosen for sequencing analysis. It was very interesting that the twenty sequenced phage clones from hIgG and hIgG1-Fc selection populations displayed the same combinations, all containing PA(A)-PG with different linking peptides (Table 1). Interesting results were also found about the distribution of random linking peptides. The different linking peptides showed divergent distribution in hIgG and hIgG1-Fc fourth post-selection populations. Of six different linking models, only PA(A)-PG (the second column in Table 2) existed in both hIgG and hIgG1-Fc post-selection populations, the other five PA(A)-PG combinations (from the third to seventh columns in Table 2) with different linking peptides existed in hIgG or hIgG1-Fc post-selection population.

**Table 1:** Sequence analyses of inserted fragments on phage clones in the original library and the third or fourth post-selection libraries with four Ig molecules

*Phage Libraries*	*Composition of single domains of inserted fragment*
The original phage library (5*)	PG-PL_9N_; PA(A)-PG-PL_6N_; PG_3N_^R^-PG^R^-PA(D)_6N_; PL_3N_-PA(D); PG_6N_-PA(A)_3N_-PL^R^
The 4th round of selection with hIgG (10)	PA(A)-PG_9N_(5**)_;_PA(A)-PG(2); PA(A)-PG_3N_(2); PA(A)_6N_-PG_3N_
The 4th round of selection with hIgG1-Fc (10)	PA(A)-PG(7); PA(A)_6N_-PG(2); PA(A)_3N_-PG-PA(A)
The third round of selection with hIgM (11)	PA(A)-PL-PL_9N_(4); PA(A)_6N_-PL_9N_(2); PA(A)-PL_3N_(3); PA(A)_9N_-PL_3N_; PA(A)_9N_-PL
The 4th round of selection with hIgA (10)	PA(A)-PL-PL_9N_(5); PA(A)_6N_-PL_9N_(2); PA(A)-PL_3N_(2); PA(A)_9N_-PL_3N_

*: Number of sequenced phage clones; **: number of phage clones with the same inserted fragment; PA(A) and PA(D): A and D domains of protein A; PG: B2 domain of protein G; PL: B3 domain of protein L; 3N, 6N and 9N: the sequence of random linking peptides composed of three, six and nine nucleotides; R: reverse sequence of original sequence.

**Table 2:** Sequences of random linking peptides in PA(A)-PG structure and their distribution in hIgG and hIgG1-Fc selected libraries

*Combinatorial form of single domains*	PA(A)-PG	PA(A)-PG_9N_	PA(A)-PG_3N_	PA(A)_6N_-PG_3N_	PA(A)_6N_-PG	PA(A)_3N_-PG-PA(A)
*Nucleotide sequence of random linking peptide*	-	*AGC TTA CAC*	*CAC*	ACC TCG *ACC*	CAC TCA	CCA
*Amino acid sequence of random linking peptide*	-	*S L H*	*H*	T S *T*	H S	P
Number of clones Selected with hIgG (10*)	2	5	2	1	0	0
*Number of clones Selected with hIgG1-Fc (10)*	7	0	0	0	2	1

*: Number of sequenced phage clones; Underlined and italic parts represent the nucleotide and amino acid sequences of linking peptide followed the second domain. 3N, 6N and 9N: the sequence of random linking peptides composed of three, six and nine nucleotides. Single letter abbreviations of amino acids: H, His; L, Leu; P, Pro; S, Ser; T, Thr.

Similar to the situation in hIgG or hIgG1-Fc selection, the displayed molecules in hIgA fourth post-selection population showed the same combinations with that in hIgM third post-selection population, all containing the PA(A)-PL (Table 1). In the twenty one randomly picked sequenced clones, twelve displayed PA(A)-PL and nine displayed PA(A)-PA(A)-PL (Table 1). In contrast with the results of hIgG and hIgG1-Fc selection populations, the sequences of linking peptides among PA(A)-PL structures tended to show convergent distribution. Almost all (4 of 5) combinations with different linking peptides existed in both hIgA and hIgM selection populations (from the second to fifth columns in Table 3), with an exception of PA(A)_9N_-PL (the sixth column in Table 3).

**Table 3:** Sequences of random linking peptides in PA(A)-PL structure and their distribution in hIgM and hIgA selected libraries

*Combinatorial form of single domains*	PA(A)-PL-PL_9N_	PA(A)_6N_-PL_9N_	PA(A)-PL_3N_	PA(A)_9N_-PL_3N_	PA(A)_9N_-PL
*Nucleotide sequence of random linking peptide*	*TAC TGG TTG*	AAA CTA *GCT AAC AAC*	*TTG*	GGT GAG ATG *CAC*	GAC TTT ATT
*Amino acid sequence of random linking peptide*	*Y W L*	K L *A N N*	*L*	G E M *H*	D F I
Number of clones selected with hIgM (11*)	4	2	3	1	1
*Number of clones selected with hIgA (10)*	5	2	2	1	0

*: Number of sequenced phage clones; Underlined and italic parts represent the nucleotide and amino acid sequences of linking peptide followed the second domain. 3N, 6N and 9N: the sequence of random linking peptides composed of three, six and nine nucleotides. Single letter abbreviations of amino acids: A, Ala; D, Asp; E, Glu; F, Phe; G, Gly; H, His; I, Ile; K, Lys; L, Leu; M, Met; N, Asn; W, Trp; Y, Tyr.

### The Ig binding properties of the representative phages

**Figure 3:**
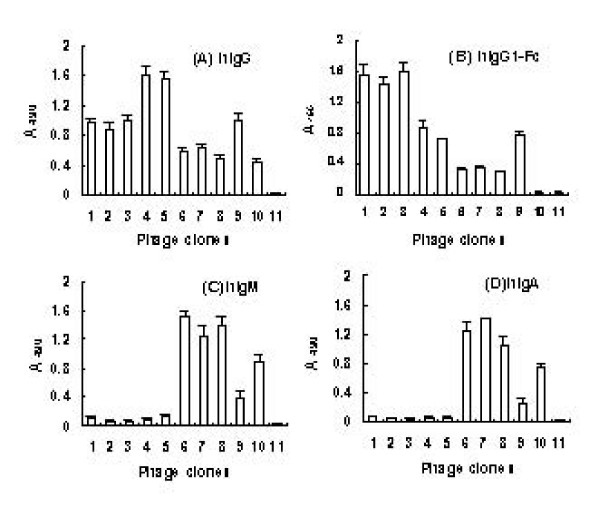
**Detection of the binding activity of representative phage clones with four Ig molecules respectively by ELISA (A-D)**. Each of Ig molecules (labeled on top of each graph) was coated on ELISA plates using 0.1 M NaHCO_3 _(pH 9.6). The amplified representative phages (2 × 10^11 ^TU) were added to each well and Ig-bound phages were detected with horseradish peroxidase (HRP)-conjugated anti-M13 antibody. The displayed sequence of each representative phages was: 1, PA(A)_3N_-PG-PA(A); 2, PA(A)_6N_-PG; 3, PA(A)-PG; 4, PA(A)-PG_9N_; 5, PA(A)_6N_-PG_3N_; 6, PA(A)_9N_-PL_3N_; 7, PA(A)-PL-PL_9N_; 8, PA(A)_9N_-PL; 9, SpA-phage (positive control); 10, 2L-phage (positive control); 11, pCANTAB5S-phage (negative control). Comparable data were obtained in three independent experiments.

Eight representative positive phage clones were chosen and tested for the Ig binding properties. As figure 3 shows, number 1 to 5 of phage clones displaying PA(A)-PG as well as SpA-phage (clone 9) possessed strong binding activity with hIgG and hIgG1-Fc, but showed little binding to hIgM or hIgA, though this binding may theoretically exist through PA(A) domain interacting with VHIII region. It was very interesting that clone 4 and 5, which were from hIgG post-selection population, showed even stronger binding to hIgG than SpA-phage as well as clone 1, 2 and 3 which were found in hIgG1-Fc post-selection population (Fig. 3A). Clone 1, 2 and 3 showed even stronger binding to hIgG1-Fc than SpA-phage as well as clone 4 and 5 which were found in hIgG post-selection population (Fig. 3B). This binding priority suggested that the linking peptides could affect the Ig-binding properties. Compared with SpA-phage and 2L-phage (clone 10), clone 6, 7 and 8 displaying PA(A)-PL from hIgM or hIgA post-selection populations showed remarkable enhanced binding to hIgM or hIgA (Fig. 3C, 3D) and weak binding activity with hIgG and hIgG1-Fc (Fig. 3A, 3B). As SpA-phage, clone 1 to 5 phages showed some weak binding to hIgM or hIgA. 2L-phage bound to hIgG, hIgM or hIgA obviously, but showed no binding to hIgG1-Fc.

### The Ig binding properties of the novel combinatorial molecules

**Figure 4:**
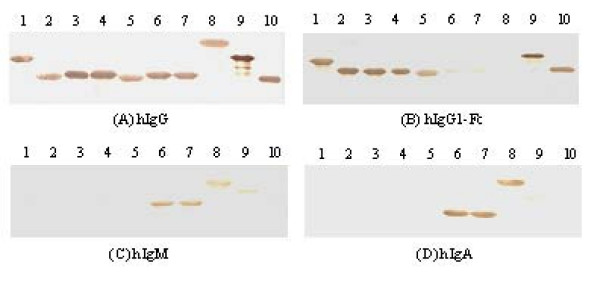
**Binding activities of seven fusion proteins of the novel combinatorial molecules, SpA, SpG and 4L with hIgG (A), hIgG1-Fc (B), hIgM (C) and hIgA (D) respectively by Western Blot**. Seven fusion proteins, SpA, SpG and 4L (each of 5 μg) were separated by electrophoresis in SDS-PAGE and electrotransferred to nitrocellulose membrane respectively. The membrane was incubated with biotin labeled hIgG, hIgG1-Fc, hIgM and hIgA in 1:3 000 dilution respectively and detected with HRP-conjugated streptavidin, followed by developing with DAB. 1, fusion protein PA(A)_3N_-PG-PA(A); 2, fusion protein PA(A)_6N_-PG; 3, fusion protein PA(A)-PG; 4, fusion protein PA(A)-PG_9N_; 5, fusion protein PA(A)_6N_-PG_3N_; 6, fusion protein PA(A)_9N_-PL_3N_; 7, fusion protein PA(A)_9N_-PL; 8, fusion protein 4L; 9, SpA; 10, SpG.

To test the binding properties of selected combinatorial molecules, these molecules were expressed as fusion proteins with thioredoxin using expression vector pET32a(+) and performed Western Blot. All the PA-PG combinations as well as SpA and SpG showed strong binding to hIgG (Fig. 4A) and hIgG1-Fc (Fig. 4B), and weak binding to hIgM (Fig. 4C) and hIgA (Fig. 4D). Inconsistent with phage binding assays, the expressed PA-PG combinations did not show any binding advantage to hIgG (Fig. 4A) and hIgG1-Fc (Fig. 4B) compared with SpA and SpG. In contrast to PA-PG, the expressed PA-PL combinations showed much stronger binding to hIgM (Fig. 4C) and hIgA (Fig. 4D) than 4L and SpA respectively, which was consistent with phage binding assay.

### The PA(A)-PG or PA(A)-PL combinations showed binding advantages

**Figure 5:**
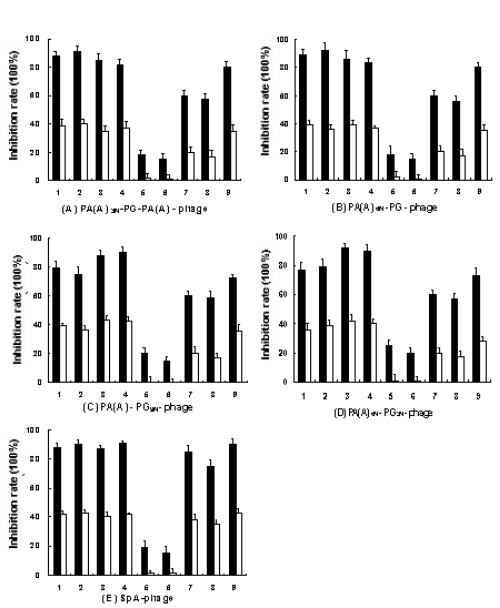
**Competitive inhibition of PA(A)-PG-phages (A-D) and SpA-phage (E) binding to hIgG by PA(A)-PG combinations, PA(A)-PL combinations, SpA, SpG and both SpA plus SpG**. 10^9^TU of tested phages without and with each of 100 nM (black bars) or 25 nM (white bars) of inhibitor proteins were added into hIgG-coated wells respectively. Unbound phages were removed and 10 μl of exponentially growing *E. coli *TG1 was added into each well, incubated for 1 h at 37°C. The TG1 cells were harvested respectively and spread LB plates containing 100 μg/ml ampicillin, and bacterial colonies were counted after incubating at 37°C overnight. Inhibition rate was calculated: [1 - (mean of the bacterial colonies from tested wells with inhibitor proteins - mean of the bacterial colonies from blank control wells) divided by (mean of the bacterial colonies from tested wells without inhibitor proteins - mean of the bacterial colonies from blank control wells)] × 100%. No. 1 to 6: The expressed fusion proteins of PA(A)_3N_-PG-PA(A), PA(A)_6N_-PG, PA(A)-PG_9N_, PA(A)_6N_-PG_3N_, PA(A)_9N_-PL_3N _and PA(A)_9N_-PL were used as inhibitors respectively. No. 7 to 9: SpA, SpG and that both were used as inhibitors respectively.

To test whether PA(A)-PG or PA(A)-PL combinations possess some binding advantages, we expressed the fusion proteins of PA(A)_3N_-PG-PA(A), PA(A)_6N_-PG from hIgG1-Fc post-selection population, PA(A)-PG_9N_, PA(A)_6N_-PG_3N _from hIgG post-selection population, and PA(A)_9N_-PL_3N _and PA(A)_9N_-PL from hIgM/hIgA post-selection population to perform competitive inhibition experiments. As figure 5 shows, all four PA(A)-PG combinations inhibited the binding of PA(A)-PG-phages to hIgG more efficiently than SpA and SpG alone (Fig. 5A, 5B, 5C, 5D). Furthermore, PA(A)-PG_9N _and PA(A)_6N_-PG_3N _from hIgG post-selection population showed more efficient inhibition than SpA, SpG and that both (Fig. 5C, 5D), while all four PA(A)-PG combinations inhibited the binding of SpA-phages to hIgG as efficiently as SpA, SpG and that both (Fig. 5E). Similar results were obtained for inhibition of PA(A)-PG-phages binding to hIgG1-Fc by competitive inhibition tests as above (data not shown). Consistent with phage binding tests (Fig. 3), the competitive inhibition experiments documented that the PA(A)-PG combinations possess some binding advantages to hIgG or hIgG1-Fc.

**Figure 6:**
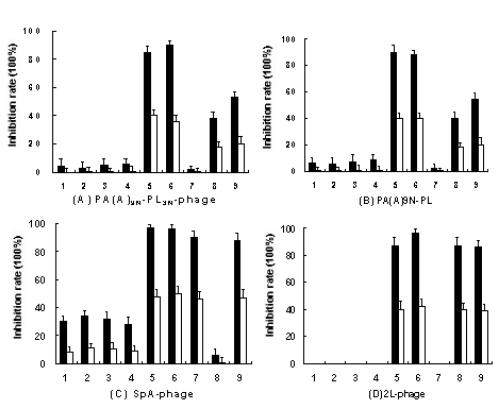
**Competitive inhibition of two representative PA(A)-PL-phages (A and B), SpA-phages (C) and 2L-phages (D) binding to hIgM molecules by PA(A)-PL combinations, PA(A)-PG combinations, SpA, 4L and both SpA plus 4L**. 10^9^TU of PA(A)-PL-phages, 10^11^TU of SpA-phages and 10^10^TU of 2L-phages without and with each of 100 nM (black bars) or 25 nM (white bars) of inhibitor proteins were added into hIgM-coated wells respectively. Unbound phages were removed and 10 μl exponentially growing *E. coli *TG1 was added into each well, incubated for 1 h at 37°C. The TG1 cells were harvested respectively and spread LB plates containing 100 μg/ml ampicillin, and bacterial colonies were counted after incubating at 37°C overnight. Inhibition rate was calculated: [1 - (mean of the bacterial colonies from tested wells with inhibitor proteins - mean of the bacterial colonies from blank control wells) divided by (mean of the bacterial colonies from tested wells without inhibitor proteins - mean of the bacterial colonies from blank control wells)] × 100%. No. 1 to 6: The expressed fusion proteins of PA(A)_3N_-PG-PA(A), PA(A)_6N_-PG, PA(A)-PG_9N_, PA(A)_6N_-PG_3N_, PA(A)_9N_-PL_3N _and PA(A)_9N_-PL were used as inhibitors respectively. No. 7 to 9: SpA, 4L and that both were used as inhibitors respectively.

For PA(A)-PL-phages competitive inhibition experiments, expressed PA(A)-PL combinations inhibited the binding of PA(A)-PL-phages to hIgM much more efficiently than 4L, SpA alone and that both (Fig. 6A, 6B), while all PA(A)-PL combinations inhibited the binding of SpA-phage (Fig. 6C) or 2L-phage (Fig. 6D) to hIgM as efficiently as SpA, SpA and 4L or 4L, 4L and SpA respectively. Similar results were obtained for inhibition of PA(A)-PL-phages binding to hIgA by competitive inhibition tests as above (data not shown). Consistent with phage binding tests (Fig. 3), the competitive inhibition experiments showed the PA(A)-PL combinations possess obvious binding advantage to hIgM or hIgA.

## Discussion

Compared with SpA-phage displaying five domains of SpA, phages displaying PA(A)-PL which was contained in each sequenced clone as predominant combinations in hIgM and hIgA post-selection populations exhibited a remarkable enhanced binding affinity for hIgM and hIgA (Fig. 3). The prokaryotic expressed PA(A)-PL combinations also showed the same binding properties (Fig. 4C, 4D). Protein L binds primarily to κ light chains of I, III, IV subtypes of Igs [8], while SpA binds about 22% hIgA and 40% hIgM through interacting with VHIII region [11,23]. The coexistence of single domains of SpA and protein L could broaden the Ig-binding spectra, and achieve the binding advantage of PA(A)-PL for hIgM and hIgA. However, the loss of other possible combinations, like PL-PL, which should have same chance to be produced in original library, and may produce enhanced affinity for κ light chains due to avidity effect, suggested that PA(A)-PL should have additional binding advantage. Considering the binding properties of protein L and SpA and the structure of Ig-Fab, we speculated that the binding advantage of PA(A)-PL might be produced through double-site binding to VHIII and Vκ regions of Fab in hIgM and hIgA. In Fab fragment of hIgM and hIgA, the conformation of VH-VL is tightly fixed due to the interchain disulfide bond between VH and VL regions and non covalent interaction of VH-VL interface [24]. Moreover, the binding sites of protein L and SpA on Ig-Fab located on the opposite surface of the antigen binding cleft, and both interactions produce little steric hindrance to each other [15,25]. These characteristics are in favor of the double site binding of PA(A)-PL to VHIII and Vκ regions. This speculation was clearly supported by results of competitive inhibition experiments which showed that 4L, SpA alone or that both couldn't inhibit the binding of PA(A)-PL-phages to hIgM or hIgA as efficiently as PA(A)-PL combinations (Fig. 6A, 6B).

It is predictable that PA(A)-PG combinations would be selected in hIgG and hIgG1-Fc post-selection populations. However, it was unexpected that PA(A)-PG was so predominant while PA(A/D)-PA (A/D) or PG-PG which had similar binding potential and same chance to be produced in original library was not selected (Table 2). This result suggests that PA(A)-PG combinations possess an advantage over other combinations in binding to Fc regions. It was supported by the phage binding assay which showed that the PA(A)-PG-phages selected by hIgG or hIgG1-Fc exhibited stronger binding to hIgG or hIgG1-Fc respectively than SpA-phages (Fig. 3A, 3B) and by the competitive inhibition test which showed that PA(A)-PG combinations inhibited the binding of PA(A)-PG-phages to hIgG or hIgG1-Fc more efficiently than SpA alone or SpG alone (Fig. 5A, 5B, 5C, 5D). The conformation of Fc was documented flexible, mobile and easy affected [26]. X-ray crystal structures studies for Fc and Fc-ligand complex indicated that the hinge proximal region of CH2 domain is disordered, suggesting internal mobility, generating a dynamic equilibrium between multiple conformers [27]. Interchange between heavy and light chain, binding to antigen and change of primary amino acid sequences of IgG (different IgG subtypes) would affect the Fc conformation [28-30]. Although SpA shares a lot of binding area in IgG-Fc with SpG, obvious difference between these two interactions was observed [31,32]. First, in SpG: Fc and SpA: Fc complex, the two helices in SpA domain are located mostly in CH2 side of Fc, the helix of SpG lies wedged in the CH2-CH3 cleft. Second, SpG interacts with Fc mainly through hydrogen bond, while SpA through hydrophobic interaction. Third, Fc has a set of unique amino acids for binding to SpG and SpA respectively [26]. So, although the binding sites of SpA and SpG overlap, their binding nature is different, and the structure of PA(A)-PG could produce the different binding avidity for a pair of Fc sites in one hIgG molecule from that produced by PA(A/D)-PA (A/D) or PG-PG, which was documented to possess some binding advantage, and therefore showed the selection advantage.

In this work, the proportion of phage clones displaying two and three domains also increased remarkably along with the rounds of selection (Fig. 1, Fig. 2), and the linking peptides were significantly selected (Table 2, Table 3). These results might reflect the effectiveness of the selection and the significance of selected PA(A)-PG and PA(A)-PL. The conformation of binding sites for IBPs of hIgA and hIgM Fab were fixed and stable, as well as the linking peptide among all selected PA(A)-PL structures showed some convergent distribution. Different from the native hIgG, the Fab of hIgG1-Fc was substituted by TNF receptor. It could produce some conformation difference between hIgG1-Fc and native hIgG, and could be responsible for the divergent distribution of linking peptide in hIgG and hIgG1-Fc post-selection populations. The phage binding assay and competitive inhibition test also showed comparable binding advantage for the clones from selection hIgG population with hIgG, and for those from hIgG1-Fc post-selection population with hIgG1-Fc (Fig. 3, Fig. 5C, 5D). This result suggested that the combinations and special linkage of the different IBP domains could sensitively reflect the conformational change in the binding sites of Ig Fc.

## Conclusion

In this study, a combinatorial phage library displaying single domain randomly-rearranged molecules derived from natural bacterial IBPs was selected with hIgG, hIgM, hIgA and hIgG1-Fc. Two kinds of novel combinations of Ig-binding domains, PA(A)-PG and PA(A)-PL, which don't exist in natural bacterial Ig-binding molecules, were obtained, and showed the comparable binding advantages. It demonstrated the novel binding properties.

## Methods

### Single domain randomly-rearranged combinatorial phage displayed library

The construction of the phage library was described previously [33]. Briefly, gene fragments encoding A and D domains of SpA [PA(A) and PA(D)], B2 domain of SpG (PG) and B3 domain of protein L (PL) were individually generated by PCR amplification using the primers (Table 4) which introduced recognition site for Xba I in both ends of the fragments and nucleoside acid sequences in the 3'-end, which encoding random linking peptide consisted of 0, 1, 2 or 3 amino acids. Then the PCR products were digested with Xba I and ligated into the Xba I site of the phagemid pCANTAB5S to construct a phage displayed random combinatorial library. The library has size of 2 × 10^7 ^members, and titer of the phage library is calculated to 1.3 × 10^11 ^transformation unit (TU)/ml. Host bacterial strain TG1 was from Stratagene Company, Cambridge, England. Primers located in the upward and downward of the cloning site of vector pCANTAB5S were used to amplify the inserted fragment of positive phages and to perform sequencing analysis of inserted fragment. Both of forward primer designated P1: 5'-CAA CGT GAA AAA ATT ATT ATT CGC-3' and reverse primer designated P2: 5'-GTA AAT GAA TTT TCT GTA TGA GG-3' was obtained from Shanghai Sangon Biological Engineering Technology & Services Co., Ltd.

**Table 4:** Primers for amplification of DNA fragments encoding each Ig-binding domains

Name	Description	Sequence(5' → 3')
PA(A)-Uxk	Sense terminal primer of PA(A)	C CTG GGT ACCTCT AGA* GCT GAC AAC AAC TTC AAC
PA(D)-Uxl	Sense terminal primer of PA(D)	TAT GGT ACC ***TCT AGA ***GCT GAC GCT CAG CAG AAC
PA(A/D)-Dxk	Antisense random primer of PA(A/D)	ACT GGT ACC ***TCT AGA ***(0N, 3N, 6N, 9N)** TTT CGG AGC CTG AGA TTC
PG-Uxk	Sense terminal primer of PG	GCG GGT ACC ***TCT AGA ***ACC TAC AAA CTG GTT ATC
PG-Dxk	Antisense random primer of PG	TCA GGT ACC***TCT AGA ***(0N, 3N, 6N, 9N) TTC GGT AAC GGT GAA GGT
PL-Uxk	Sense terminal primer of PL	GCG GGT ACC***TCT AGA ***AAA GAA AAA ACC CCG GAA
PL-Dxk	Antisense random primer of PL	TGC GGT ACC ***TCT AGA ***(0N, 3N, 6N, 9N) ACC AGC GAA TTT GAT GTT CAG

*: Italic and black parts represent Xba I recognition sites; **: Underlined parts represent the random linking sequence; 0N, 3N, 6N and 9N: the sequence of random linking peptides composed of non, three, six and nine nucleotides.

### Vectors and reagents

Phagemid vector pCANTAB5S and phage displaying E-D-A-B-C domains of SpA (SpA-phage) were constructed by our lab and has been described previously [34]. Briefly, phagemid pCANTAB5S was obtained by inserting the DNA fragment of Xba I-Stu I-Sal I-Kpn I-(Gly4Ser)3 into pCANTAB5L (Pharmacia Biotech, Uppsala, Sweden) between Sfi I and Not I cloning sites. The encoding sequence of SpA [35] was inserted into pCANTAB5S at Stu I site to construct SpA-phage. 2L-phage containing two domains (B3-B3) of protein L was obtained from a phage library displaying Ig-binding mono-domains of SpA and protein L by affinity selection with hIgG [22]. Human IgG (hIgG), human IgM (hIgM), human IgA (hIgA) and SpG were from Sigma, St. Louis, MO, USA. Prokaryotic expressed SpA (Genbank: P02976) and hIgG1-Fc molecule that is obtained through substituting Fab of human IgG1 with soluble receptor of human tumor necrosis factor (TNF) by gene engineering were kindly provided by Shanghai Fudan-Zhangjiang Bio-Pharmaceutical Co. Ltd, Shanghai, China. All antibodies were biotinylated using biotinyl-N-hydroxy-succinimide (Pierce, Rockford, IL, USA). Purified protein 4L containing four domains (B3-B3-B3-B3) of protein L was expressed by using prokaryotic expression vector pET32a(+) in *E. coli *BL21 following the protocol provided by Novagen Company (Germany) and purified by Ni-NTA column (Pharmacia Biotech) at our lab (data not shown). Helper phage M13K07 and horseradish peroxidase (HRP)-conjugated anti-M13 antibody were from Pharmacia Biotech, Uppsala, Sweden.

### Selections of the phage displayed library with four Ig molecules

hIgG, hIgM, hIgA and hIgG1-Fc molecules were diluted in coating buffer (0.1 M NaHCO_3_, pH 9.6) resulting in 10 μg/ml respectively and coated in sterile 96-well ELISA plates at 37°C for 3 h. After blocking the plates with blocking buffer (10% degreased milk powder, 0.1% Tween 20 and 0.2% mercurothiolate in 0.01M phosphate-buffered saline) for at least 1 h, phage displayed library (about 10^10–11 ^TU) was added into each well and incubated for 3 h at 37°C. Unbound phages were removed by washing with washing buffer (0.25% Tris, 0.05% Tween 20 in ddH_2_O) 30 times with vigorous pipetting, and 100 μl *E. coli *TG1 at an optical density at 600 nm of about 0.5 were added into wells, incubated for 1 h at 37°C. The number of eluted phages was calculated by colony forming units (c.f.u.) on TG1 cells in LB plates containing 100 μg/ml ampicillin. *E. coli *TG1 cells harboring eluted phage were amplified for 1 h by shaking 250 rpm at 37°C in 8 ml LB medium. Then ampicillin (100 μg/ml) and helper phages M13K07 (about 3 × 10^12 ^TU) were added and the *E. coli *TG1 cells were cultured as above. After 1 h, kanamycin (50 μg/ml) was added and the *E. coli *TG1 cells were grown continuously by shaking 180 rpm at 37°C overnight. Phages were harvested by centrifugation (10 min, 5000 × g) of the medium and filtration of supernatant through 0.22 μm filter membrane, then used for the subsequent round of selection with the same bait. Three or four rounds of selection were performed as above.

### Detection of distribution and size of inserted fragments in primary library and each round post-selection population by PCR

Twenty two phage clones in primary library and each round post-selection population were picked randomly and cultured in 0.5 ml LB medium by shaking 250 rpm at 37°C for 5 h respectively. The culture medium was used as template to amplify inserted fragments in these phages by PCR. For DNA amplification, 1 μl of template was added to a 50 μl reaction mixture containing 5 μl of 10× reaction buffer (500 mM KCl, 100 mM Tris-HCl pH 9.0, 1% Triton X-100), 1 μmol of each primer (P1 and P2), 3 mmol Mg^++^, 100 μmol dNTP, 1 U Taq DNA polymerase (2 U/μl, Promega, Madison, WI, USA) and nuclease-free water. The reaction mixture was amplified on a thermocycler (Perkin Elmer Applied Biosystems, USA) for 30 cycles of 30 s at 94°C, 30 s at 50°C, and 45 s at 72°C followed by a 5 min extension at 72°C. PCR products were analyzed by electrophoresis in 1.2% agarose gel and detected by staining with ethidium bromide. pCANTAB5S phagemid and blank culture medium were used as template for positive and negative controls respectively.

### Sequence analyses

Five to eleven positive phage clones identified by PCR were picked randomly from the primary phage library and the third or fourth post-selection populations. Inserted DNA fragment of positive phages were sequenced using the primers P1 and P2. Corresponding amino acid sequences were deduced from DNA sequences and a multiple sequence alignment was analyzed with the DNASTAR software package.

### Detection of representative positive phages binding to Ig molecules by ELISA

10 μg/ml each of Ig molecules (hIgG, hIgM, hIgA or hIgG1-Fc) was coated on ELISA plate as described above. About 2 × 10^11 ^TU amplified representative positive phages obtained from the each round of selection were added to each well and then incubated for 2 h at 37°C. The wells were washed with phosphate-buffered saline (PBS) containing 0.05%Tween 20 and the bound phages were detected with HRP-conjugated anti-M13 phage antibody. The development was performed by the addition of diaminobenzidine (DAB) (Sigma, St. Louis, MO, USA), and read at 490 nm in an ELISA Reader (Bio Rad). SpA-phages and 2L-phages were used as positive controls respectively. The pCANTAB5S-phage (obtained by infecting *E. coli *TG1 with blank phagemid pCANTAB5S) was used as a negative control.

### Expression of the novel combinatorial molecules

Bacterial clones harboring positive phagemids displaying five PA(A)-PG combinations and two PA(A)-PL combinations were used as template respectively to amplify DNA fragments by PCR using forward primer (5SNco-u) and reverse primer (5SNoG-d). The forward primer 5SNco-u contained Nco I recognition site (Table 5). The synthetic primers were obtained from Shanghai Sangon Biological Engineering Technology & Services Co., Ltd. For DNA amplification, the composition in 50 μl reaction mixture was the same as above. The reaction mixture was amplified on a thermocycler (Perkin Elmer Applied Biosystems, USA) for 35 cycles of 30 s at 94°C, 30 s at 60°C, and 45 s at 72°C followed by a 5 min extension at 72°C. The amplified DNA fragment containing a BamH I cloning site of the phagemid at 3' terminal of displayed sequence was digested with Nco I and BamH I and inserted into the Nco I-BamH I site of prokaryotic expression vector pET32a(+) (Qiagen, Valencia, CA). The recombinant plasmid was identified and sequenced by forward sequencing primer (B-S-U) and reverse sequencing primer (S-H-D) (Table 5). Competent *E. coli *BL21(DE3) cells were transformed using above positive recombinant plasmid mediated by CaCl_2 _and spread on LB plates (containing 100 μg/ml of Amp and 15 μg/ml of Kana), and cultured at 37°C overnight. Transformed positive BL21(DE3) colony was picked up and cultured in 500 ml LB medium by shaking 250 rpm at 37°C with ampicillin (100 μg/ml). The Log-phase bacteria were induced expression by adding 500 μl of 1 M isopropyl-beta-D-thiogalactopyranoside (IPTG) in the medium and continuously cultured for 3 h. The pellet cells were collected after centrifugation at 6 000 rpm for 10 min at 4°C and washed by PBS (pH 7.2). Then the cells were resolved by using 8 M urea (pH 8.0) and Ni-NTA column (Amersham Pharmacia Biotech) was used to purify the expression proteins. The purified proteins were dialyzed thoroughly against PBS (pH 7.0). The concentrations of the proteins were detected by routine Bradford assay.

**Table 5:** Primers for amplifying exogenous DNA sequences of selected representative phages

*Name*	*Description*	*Sequence (5' → 3')*
5SNco-u	Forward amplifying primer	TATCCATGG*CTGCGGCCCAGCCGGCCTCT
5SNoG-d	Reverse amplifying primer	CCTGCGGCCGCAACTGCCGCCGCC
B-S-U	Forward sequencing prime	GGA TCC GAG CTC AGG CCT GTC GAC GGT ACC GTT
S-H-D	Reverse sequencing primer	GAG CTC AAG CTT ACC AGA TCC ACC ACC GCC GGT ACC

*: Underlined part represents Nco I recognition site.

### Competitive inhibition test

In order to avoid experimental results being interfered due to binding of SpA-phage and other phages displaying more than two IBP domains to conjugated secondary antibodies, the competitive inhibition tests were established in this study by using *E. coli *TG1 infection as a substitute for HRP-conjugated anti-M13 phage antibody detection. Briefly, 1 μg each of Ig molecules (hIgG, hIgG1-Fc, hIgA or hIgM) were coated on sterile 96-well microtitration plates by using 0.1 M NaHCO_3 _(pH 9.6) at 37°C for 3 h. After blocking the plates with blocking buffer (10% degreased milk powder, 0.1% Tween 20 and 0.2% mercurothiolate in 0.01 M phosphate-buffered saline) for at least 1 h, the tested phages without and with 100 nM or 25 nM of inhibitor proteins were added into hIgG-coated or hIgG1-Fc-coated wells respectively, and incubated at 37°C for 1 h. Unbound phages were removed by washing with washing buffer (0.25% Tris, 0.05% Tween 20 in ddH_2_O) 10 times with vigorous pipetting after incubating for 3 h at 37°C, and 10 μl *E. coli *TG1 at an optical density at 600 nm of about 0.2 was added into each well, incubated for 1 h at 37°C. The infected TG1 cells of each well were harvested respectively and spread LB plates containing 100 μg/ml ampicillin, and bacterial colonies were counted after incubating at 37°C overnight. Three parallel wells of each test were detected, and the mean of bacterial colonies from each test wells was used to calculate the inhibition rate. Inhibition rate wascalculated: [1 - (mean of the bacterial colonies from tested wells with inhibitor proteins - mean of the bacterial colonies from blank control wells) divided by (mean of the bacterial colonies from tested wells without inhibitor proteins - mean of the bacterial colonies from blank control wells)] × 100%.

### Western Blot

Each of 5 μg of the tested combinatorial IBP molecules, SpA, SpG and 4L were separated by electrophoresis in sodium dodecyl sulphate-polyacrylamid gel electrophoresis (SDS-PAGE) and electrotransferred to nitrocellulose membrane (Millipore, Pharmacia) respectively. The membrane was blocked with blocking buffer (10% degreased milk powder, 0.1% Tween 20 and 0.2% mercurothiolate in 0.01 M PBS) at 4°C over night. After washing with PBS containing 0.05% Tween 20, the membrane was incubated with labeled hIgG (1 mg/ml, 1:3 000) at 37°C for 2 h. The membrane was washed with PBS containing 0.05% Tween 20 for 6 times and detected with HRP-conjugated streptavidin, followed by developing with diaminobenzidine (DAB). The interactions of the tested proteins with hIgG1-Fc, hIgM or hIgA were parallel detected by Western Blot respectively as above.

## Authors' contributions

HY, JC and L–QL carried out the selections of the phage displayed library with four Ig molecules and drafted the manuscript. XZ, Q–LC and Z–MW performed the detection of distribution and size of inserted fragments in primary and each round post-selection population by PCR, and competitive inhibition test. HY and W–TL carried out expression of the fusion proteins and Western blot experiments. S–HJ, RX, J–AJ and XP performed the ELISA of selected positive phages and the sequence analyses. WP and Z–TQ conceived, designed and coordinated the original project. WP and JC wrote and revised the manuscript. All authors read and approved the final manuscript.
